# Financial Leverage, Economic Growth and Environmental Degradation: Evidence from 30 Provinces in China

**DOI:** 10.3390/ijerph17030831

**Published:** 2020-01-29

**Authors:** Miyun Zhao, Rui Yang, Yi Li

**Affiliations:** 1 School of Economics and Management, Chang’an University, Xi’an 710064, China; liyi@chd.edu.cn; 2 Department of Science and Technology, Chang’an University, Xi’an 710064, China; Lyyangrui@chd.edu.cn

**Keywords:** financial leverage, economic growth, carbon emissions, GMM panel VAR

## Abstract

This study seeks to investigate the endogenous relationship between financial leverage, economic growth and environmental degradation in China by employing a the generalized moments method (GMM) panel vector autoregressive (PVAR) approach with a panel of data from China’s 30 provinces over the period 1997–2016. Three key results arise. First, financial leverage can significantly lessen economic growth, while economic growth decreases financial leverage. Second, economic growth provides an important impetus to boost carbon emissions. Finally, carbon emissions have inversely pushed up financial leverage. These results reflect to some extent China’s impressive rate of economic growth, which has been attained via continuously supporting inefficient state-owned enterprises and heavy and polluting industries through bank loans. The results are further supported by the variance decomposition. The findings provide valuable policy implications for deepening financial supply-side structure reform to transform and upgrade China’s real economy. These policy implications are conductive to developing a low-carbon economy.

## 1. Introduction

Since the global financial crisis of 2008, there has been upsurge in the awareness of financial leverage and its harmful impacts on economic growth among excessive debt accumulation and financial instability. China was heavily influenced by the current global financial crisis, resulting in a significant increase in its financial leverage ratio, while economic activity declined. The Chinese government adopted a 4 trillion yuan ($586 billion) stimulus package, which was announced on 9 November, 2008, to boost economic growth. However, China has been unable to continue the rapid output growth that occurred before the recession. Instead, China has entered into a new normal and faced mounting downward economic pressure. Moreover, China’s financial leverage has strongly increased from 149% in 2008 to 175% in 2009, over a short period of stability from 2009 to 2011, and then rapidly trended up to reach 180% in 2012. That trend has kept increasing at an unabated pace and has broken new highs, up to 208%, in 2016. There has been some financial deleveraging, albeit slow, since 2016 in China, while leaving the ratio at elevated levels, which implies that China has entered an age of unprecedented slower nominal growth and high financial leverage.

Additionally, in 2006, China became the largest emitter of energy-related carbon dioxide (CO_2_) emissions in the world and has kept this position thereafter. In recent years, haze weather has become an alarming concern in many cities. Pollution prevention and control was declared to be one of the three critical battles by the 19th National Congress of the Communist Party of China. Meanwhile, the Chinese financial system, dominated by the banking sector, has played a remarkable role in making credit available to inefficient state-owned enterprises, benefitting from the policies enacted by the government in China. Over the past years, this preferential treatment has led to inefficient allocation of financial resources for heavy and polluting industries [1]. Therefore, to some extent, China’s impressive rate of economic growth has been attained at the cost of the environment and the support of bank loans and financial leverage.

The linkages between economic growth and environmental degradation have attracted particularly significant concern in academic and policy circles. While there is evidence that a decrease in CO_2_ emissions is accompanied with the process of economic growth, for China it remains unclear. Recently, the relationship of financial leverage and environmental degradation has aroused considerable concerns, as financial leverage that is too high increases credit use in low efficiency state-owned enterprises, which may further hinder growth in the economy and increase the burden on environment. According to the previous studies, banking lending plays a major role in transitioning to a low-carbon society [2]. In contrast, He et al. argues that bank lending is much more abundant in heavy and pollution industries, and so this unbalanced bank lending will harm the environment and economic growth [3]. Furthermore, when increasing financial resources go to heavy industries, financial development leads to more environmental ills [4] and the purely profit-maximization decisions of financial institutions will cause high environmental costs of economic growth [1]. Therefore, it is important to demonstrate whether financial leverage ratio can leverage economic growth and environmental governance and clarify how financial leverage affects the growth and environment. Meanwhile, these problems are also important for countries and regions facing the same phenomenon. The purpose of this present study is to explore the linkage among financial leverage, economic growth and CO_2_ emissions. We aim to discover the logical mechanism among high financial leverage, accelerated economic downturn and prominent environmental problems in China, and the interaction between the financial leverage, economic growth and environmental degradation.

The remainder of this paper is organized as follows. Section 2 reviews the related literature on the nexus of financial leverage, economic growth and environmental degradation. Section 3 introduces the methodology and description of variables used in this research study. Section 4 presents and discusses the empirical results in detail. In the last section, we conclude and propose policy implications.

## 2. Literature Review

Schumpeter established a so-called finance-growth nexus by highlighting the contribution of the financial system to the growth in the economy [5]. Financial intermediaries not only identify the best production technologies, but also boost the rate of technological innovation. This can improve resource allocation and risk amelioration and thereby accelerate economic growth. King and Levine reckoned that a well-developed and solid financial system matters for economic growth [6]. In particular, from the financial deepening theory and debt-deflation theory perspectives, tow propositions are particularly interesting: (1) the positive influence of financial leverage on economic growth is due to financial deepening and development that causes economic growth. Levine illustrated that the development of finance can boost economic growth by promoting capital allocation, improving corporate governance, controlling risks and facilitating transactions, and credit growth can exert positive effects on economic development through the income effect and the investment effect [7]. (2) Financial leverage adversely affects growth in the economy. Fisher developed a so-called debt-deflation theory, stressing that a rising financial leverage ratio will hinder economic growth [8], especially when debt accumulates to a level that may lead the economy into a vicious spiral of debt deflation, so as to trigger a recession. Rousseau and Wachtel pointed out that excessively rapid growth of credit or excessive financial deepening can weaken banking systems, which in turn will give rise to economic growth-inhibiting crises [9]. Schularick and Taylor demonstrated that credit growth and increasing financial leverage are powerful predictors of financial crises, and hence cause drag on real economic gains [10]. Ouyang and Li found that financial development has a significantly inhibitory effect on economic growth in China [11].

Furthermore, since the seminal work that established the growth–environment link by Grossman and Krueger [12], a plethora of studies were done on the association between economic growth and environmental degradation. However, the conclusions are not consistent and can be categorized basically into three cases. First, far from bringing ever-greater threat to the environment, growth in the economy appears to sow the seeds of maintaining and improving environmental quality. Tamazian et al. revealed that more economic growth lowers environmental dilapidation [13]. Second, regarding the adverse environmental effects of economic growth, some have asserted that higher levels of economic development entail more pollution and pressure on the environment. In fact, the economy is able to grow while improving environmental quality because of technological progress in abatement [14]. Third, an inverted U-shaped relationship exists among growth and the environment, called the Environmental Kuznets Curve (EKC), which was inspired by Grossman and Krueger [15]. The EKC hypothesis confirms that growth in the economy is first followed by worsening and then improving the environment, and this hypothesis shows that both economic scale and development degree are good for the environment in the long run. As for China, a previous study indicated that growth in the Chinese economy is closely connected with carbon emissions. For instance, over the last decade, economic growth has acted as a significant driver for an increase in CO_2_ emissions [16].

In fact, environmental degradation does not necessarily depend on the economic growth level alone; financial leverage may be another source. The common measure of financial development, the financial leverage ratio, equals the value of credit or money divided by the GDP [7]. From this perspective, the bulk of existing research suggests that financial leverage is an efficient strategy to improve environmental quality. As Tamazian et al. rightly pointed out, more financial system development and openness in Brazil, Russia, India and China (BRIC) economies, attracting a higher degree of research and development-related foreign direct investment (FDI), which props up technological innovations and results in energy efficiency, helps to lower carbon emissions and develop the financial sector. This results in allocating financial resources [13], reducing financial risk and borrowing costs, and encouraging investment activities for environment-associated projects [17]. Furthermore, Jalil and Feridun mainly examined the finance–environment nexus in China [18]. They found that financial development fosters an important decline in carbon emissions suggesting that China’s financial development has not taken place at the cost of environmental degradation. Shahbaz et al. confirmed that the development of finance, measured by the per capita access to domestic credit in the private sector, is a major contributor to lessening carbon emissions in the South African economy [19].

However, there are also some studies that oppose the arguments above. For example, Zhang found that China’s financial leverage, the ratio of loans in financial intermediation to GDP, gives an important impetus to boost carbon emissions [20]. Javid and Sharif documented that the development of finance significantly promotes the increase of environmental degradation, while Shahbaz et al. argued that bank-based financial systems adversely affect the environment [21,22]. Similarly, Pata showed that financial development is one of the top three factors causing increases in CO_2_ emissions in Turkey [23]. Khan revealed that the development of finance positively affects environmental pollution, while increasing financial resources are allocated to non-green industries [24]. For China, Yin et al. documented that financial development of big cities is the impetus to increase the burden on both air and water quality [25]. In particular, Shahbaz confirmed that financial instability increases environmental degradation in Pakistan [26].

Although the aforementioned studies regarding the nexus of finance-growth, growth-environment and finance-environment have obtained plentiful results, there are certain areas that are worth further study. First, the available literature investigates either the effect of financial deepening and development or the impact of financial instability on economic growth and environmental quality; few studies combine the two to give a comprehensive analysis. To our knowledge, the dynamics of financial leverage, economic growth, and environmental degradation have not been studied across a sample of China’s 30 provinces over the long run. In addition, unlike previous studies that have always applied the univariate class of models, this study will explore the leverage–growth–environment nexus for China in a multivariate approach known as the PVAR model, which allows us to introduce the specific fixed and time effects simultaneously. The results will be more accurate and reliable, and thereby more in line with reality.

Following in the footsteps of previous research, the current study aims to contribute in two ways: (1) Regarding financial leverage, this study combines financial deepening and development and financial instability to examine the relationship between the leverage, growth and environment of China’s 30 provinces. (2) This study adopts a recent multivariate econometric tool, the PVAR technique, including the impulse response function tool and variance decomposition analysis, to better understand the dynamic interaction of three variables of interest, financial leverage, economic growth and carbon emissions in China.

## 3. Methodology and Data

### 3.1. Methodology

The PVAR method, a proper methodology to explore macroeconomic dynamics, was first developed by Holtz-Eakin et al. [27], and then adopted by Love and Zicchino [28]. The PVAR approach combines the panel-data approach and the traditional VAR model, which has several practical benefits. First, the PVAR model is neutral with regards to a particular theory. Second, all variables in a PVAR system are mutually treated as endogenous, which is in line with the interdependence realities. Third, PVAR allows for unobserved individual heterogeneity.

The PVAR model for our paper is specified as follows:

(1) $$Y_{i,t}=A_{0}+A\left(L\right)Y_{i,t}+u_{i}+\delta_{i,t}+\varepsilon_{i,t}$$

where $Y_{i,t}$ is a 3-dependent-variable vector $\left\{LEV,\;GDP,\;CO_{2}\right\}$, LEV, GDP and $CO_{2}$ are financial leverage ratio, economic growth and carbon emissions respectively. Let i be index province and t time. $A_{0}$ is the constant vector. A(L) indicates the matrix polynomial in the lag operator with the coefficient vector to be estimated, $A\left(L\right)=A_{1}L^{1}+A_{2}L^{2}+\cdots +A_{p}L^{p}$. $u_{i}$ represents province-specific fixed effects or the individual heterogeneity of each province, which accounts for the time invariant individual effects unobserved at the province level, and $\delta_{i,t}$ indicates province-specific time effects in order to capture any macro shock that may affect all provinces correspondingly. $\varepsilon_{i,t}$ denotes random disturbance with $E\left(\varepsilon_{i,t}\right)=0$, $E\left({\varepsilon^{\prime }}_{i,t}\varepsilon_{i,t}\right)=\Sigma$, and $E\left({\varepsilon^{\prime }}_{i,t}\varepsilon_{i,j}\right)=0$ for t > j.

Because the lag term of the explained variables $Y_{i,t}$ is incorporated in the specification, the province fixed effects indicated by $u_{i}$ are associated with the regressors. Using the standard mean-differencing method to remove the fixed effect would generate biased and subjective coefficients. Hence, we use forward mean-differencing, which is explicitly the ‘Helmert procedure’ [29], to preserve the orthogonality among transformed and lagged explanatory variables and allow the lagged regressors that are used as instruments to more consistently estimate the coefficients, adopting the system of GMM. Furthermore, treating the endogenous variables as first-difference with the optimal autoregressive lag order j and no constant, the PVAR model reported in the regression (1) can also be described as:

(2) $$\Delta \left(LEV_{i,t}\right)=\sum_{j=1}^{p}a_{1j}\Delta \left(LEV_{i,t-j}\right)+\sum_{j=1}^{p}b_{1j}\Delta \left(GDP_{i,t-j}\right)+\sum_{j=1}^{p}c_{1j}\Delta \left(CO_{2}{}_{i,t-j}\right)+u_{1_{i}}+\delta_{1_{i,t}}+\varepsilon_{1_{i,t}}$$

(3) $$\Delta \left(GDP_{i,t}\right)=\sum_{j=1}^{p}a_{2j}\Delta \left(LEV_{i,t-j}\right)+\sum_{j=1}^{p}b_{2j}\Delta \left(GDP_{i,t-j}\right)+\sum_{j=1}^{p}c_{2j}\Delta \left(CO_{2}{}_{i,t-j}\right)+u_{2_{i}}+\delta_{2_{i,t}}+\varepsilon_{2_{i,t}}$$

(4) $$\Delta \left(CO_{2_{i,t-j}}\right)=\sum_{j=1}^{p}a_{3j}\Delta \left(LEV_{i,t-j}\right)+\sum_{j=1}^{p}b_{3j}\Delta \left(GDP_{i,t-j}\right)+\sum_{j=1}^{p}c_{3j}\Delta \left(CO_{2}{}_{i,t-j}\right)+u_{3_{i}}+\delta_{3_{i,t}}+\varepsilon_{3_{i,t}}$$

The panel impulse response functions (IRFs) describe the reaction of one variable in response to the variations in another variable in the system when all other shocks are kept equal to zero. Moreover, to examine the IRFs, it is especially important to determine an appropriate order of variables in the system [30]. As is known to all, the identifying assumption is that the variables that come earlier in the systems are more exogenous, while the ones that come later are more endogenous [28]. In our specification, we assume that financial leverage appears earlier than economic growth and carbon emissions for two reasons. First, financial deepening and development measured by financial leverage is treated as a technological factor in the model of neoclassical economic growth, while carbon emissions are treated as an undesirable output. Second, this model focuses only on China, so as the shocks to financial leverage are more likely to be more exogenous. For example, the global financial crisis that originated from the USA in 2008 is a notable shock to China. Moreover, we also suppose that current shocks to the growth in the economy create an influence on carbon emissions simultaneously, or even with a lag, while the shocks to carbon emissions cause an impact on economic growth only with a lag. Thus, economic growth appears before carbon emissions in the model. In a word, the order of variables is set as: financial leverage, economic growth, then carbon emissions.

To conduct the impulse response functions analysis, we estimated the confidence intervals, 5th and 95th percentile bounds, using Monte Carlo simulations with 1000 bootstraps. In addition, we adopted the variance decomposition analysis to show the variation in percentages in a variable that are attributable to the shock to another variable accumulated over time. The variance decomposition specifies the degree of the total accumulated effect over six-yearly periods in the current paper.

### 3.2. Data

Yearly panel data from 1997 to 2016 for 30 Chinese provinces were collected from the China Statistical Yearbook, Provincial Statistical Yearbooks and China Energy Statistical Yearbook over the years of 1996–2017. Considering the availability and validity of the data, Tibet, Hong Kong, Macau and Taiwan were dropped in the research scope of this paper.

#### 3.2.1. Carbon Emissions Estimation Model

This paper adopts the carbon emissions calculation method, which is provided by the IPCC [31], to obtain carbon emissions for China’s 30 provinces. The formula can be expressed as follows.

(5) $$CO_{2}=\sum_{i=1}^{8}\left(CO_{2}\right){}_{i}=\sum_{i=1}^{8}E_{i}\times NCV_{i}\times CEF_{i}\times COF_{i}\times \frac{44}{12}$$

where $CO_{2}$ denotes carbon emissions per capita, *i* indicates fossil fuel type, *E* represents fossil fuel consumption, NCV, CEF and COF define the average low calorific value provide by the China Energy Statistical Yearbook., the carbon content, and the rate of carbon oxidation, respectively. The carbon content per unit heat and the rate of carbon oxidation of the eight fossil fuels were obtained from the Guidelines to Provincial Lists of Greenhouse Gas Inventory. The carbon emissions coefficients of various fossil fuels are presented in Table 1.

#### 3.2.2. Data Description

Three variables were used in the current study to investigate the nexus of leverage–growth–environment of China’s 30 provinces. These variables include: (1) the financial leverage ratio, measured by the credit to private sector as the share of the nominal GDP, (2) the real GDP per capita, (3) carbon emissions per capita as a determinant of environmental degradation. All variables were taken as the natural logarithm to reduce non-normality and heteroscedasticity, except financial leverage ratio before estimations. The variables description and data sources are shown in Table 2.

Similar to previous studies [11,32,33], the data of explanatory variables from 1997 to 2016 is listed in Table 3.

## 4. Results and Discussion

### 4.1. Panel Unit Root Tests

All before implementing the PVAR framework, the first step of the estimation process is to test the stationary properties of all series. In fact, two categories of the panel unit root test exist: the first methods, such as the tests of LLC, Breiitung and Hadri, assume a common unit root process of each cross-sectional series; whereas the second includes IPS, Fisher-ADF and Fisher-PP, which relax the basic hypothesis and assume an individual unit root process of each cross-sectional series. Nevertheless, there is a significant difference among the financial leverage, economic growth and environmental degradation of the 30 Chinese provinces. Hence, the individual unit root tests should be used.

The results of IPS, Fisher-ADF and Fisher-PP are similar, as reported in Table 4. All three tests calculated show strong evidence that the first difference series are integrated with the first order and most of them reject the null hypothesis of the unit roots at a 1% level of significance. This means that all of the variables in the first difference are stationary.

### 4.2. PVAR Estimation Results

To eliminate province-fixed and time effects prior to estimating the three-variable PVAR model, the GMM method was used, as shown in Table 5.

First, for the financial leverage equation, as expected, the first lag of financial leverage is positively and significantly correlated with its current level. Moreover, we found evidence for a negative effect of the first lag of economic growth on financial leverage. This result is particularly interesting because it confirms that financial deleveraging is a process that occurs with economic growth. Last, unexpectedly, carbon emissions exert a positive effect on financial leverage at a 1% level of significance, implying that an extensive economy growth, dominated by highly polluting industry, relies on massive amounts of credit aid.

Second, regarding the economic growth equation, as expected, the first and second lags of economic growth are significant, with a positive sum of coefficients equal to 1.016. Additionally, the results indicate that the second lag of financial leverage weakly negatively influences the degree of economic growth at the 5% significance level. This result clearly indicates that extensive financial leverage impedes economic growth via excessive credit accumulation.

Finally, for the equation related to the carbon emissions, the coefficient of the first lagged value of economic growth is significantly and positively associated with the level of carbon emissions. Moreover, the first and second lags of carbon emissions positively impact on their current level. Regarding the financial leverage, the result shows that it does not determine the level of carbon emissions of China’s 30 provinces, whatever the lag considered.

### 4.3. Impulse Response Functions Discussion

**Figure 1:**
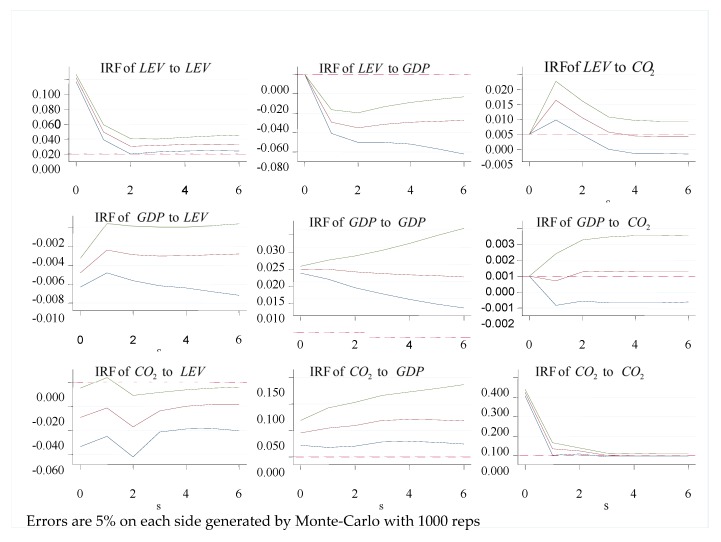
Impulse Responses for the two lag VAR of *LEV GDP CO*_2_.

This subsection reports and discusses the results from the simulations of the impulse response functions of the two-period lag three-variable VAR of financial leverage, economic growth, and carbon emissions. Figure 1 illustrates the impulse response graph for the model with three variables when one standard deviation shock is given. The 5% error bands are generated by Monte Carlo simulation with 1000 repetitions.

To avoid confusion and compare with existing studies, we synthesized the results into three categories: financial leverage and economic growth, economic growth and carbon emissions, and financial leverage and carbon emissions.

We begin with the nexus of financial leverage and economic growth. Surprisingly, a shock to financial leverage exerts a significantly negative effect on the growth in the economy due to bank sectors reducing rather than increasing resource allocation efficiency via continuously supporting low efficiency state-own enterprises in China. On the other hand, economic growth also has a negative feedback effect on financial leverage. Both of them are consistent with the PVAR regression results.

Next, we turn our attention to the association among economic growth and carbon emissions. Notably, these results imply that one standard deviation shock of economic growth produces a positive effect on carbon emissions. Because carbon emissions do not affect economic growth in the PVAR estimation, the interpretation of the influence of one standard deviation shock in carbon emissions on economic growth is conductive for understanding the expected influence if carbon emissions significantly impact economic growth with the same obtained sign. The reactions of growth in the economy to one standard deviation shock to carbon emissions are firstly negative but then positive from the second year to year 6. At the same time, the results indicate that the largest adverse effect appears in the first year with a value of −0.0003. Generally, the influence on economic growth of carbon emissions is very feeble and weak.

Last but not least, the purpose of this paper is the results of the response of carbon emissions to one standard deviation shock in financial leverage. Similar to the influence of shock in carbon emissions on economic growth, the financial leverage also does not determine carbon emissions in the PVAR estimation. The interpretation of the reaction of carbon emissions to one standard deviation shock in financial leverage benefits the explanation of the expected impact if financial leverage significantly impacts carbon emissions in the same obtained sign. The results indicate that one standard deviation shock in financial leverage negatively affects carbon emissions, and fluctuates in significance from the first year to year 3. In addition, one standard deviation shock in carbon emissions produces a significantly positive effect on financial leverage in the current period, but the reaction gradually declines and turns negative in the third period. This means that the increase in carbon emissions triggers a short-term rise in financial leverage, but accompanying economic stimulus in the future, financial leverage will be lower when a shock in carbon emissions occurs.

### 4.4. Impulse Response Functions Discussion Variance Decomposition Analysis

For specifying the magnitude and degree of the impact on other variables of change in one variable, we further apply the variance decomposition of the PVAR model, which provides information about the percentage changes in the explained variables because of their own and the other variable shocks. Table 6 presents the variance decomposition results. During the 10th forecast period, the variance decomposition indicates that financial leverage explains approximately 6.9% of the changes in economic growth and 3.5% of the variations in carbon emissions. Economic growth explains approximately 63.1% of the variations in financial leverage and 26.8% of the changes in carbon emissions. Carbon emissions explain approximately 0.5% of the changes in financial leverage but have no explanation for the fluctuations in economic growth. The last fluctuations confirm that the influence of financial leverage on the deviation of economic growth and carbon emissions are relatively small, meaning that financial leverage is less able to explain the two variables, while economic growth provides a large feedback effect for financial leverage, and the contribution of economic growth to the deviation in carbon emissions is not small. However, carbon emissions have little feedback impact on financial leverage and economic growth. In summary, economic growth has a remarkable explanation for the fluctuation in financial leverage and carbon emissions. In the long term, growth in China relies more on heavy and polluting industries with high credit demand.

## 5. Conclusions

This paper quantitatively analyzes the dynamic relationship among financial leverage, economic growth and carbon emissions of 30 Chinese provinces by applying the PVAR model. The following main conclusions are obtained.

First, financial leverage adversely influences economic growth, while economic growth also has a remarkable negative effect on financial leverage in China. On one hand, this result implies that rising financial leverage would hinder rather than boost China’s economic growth. In line with Ouyang and Li [11], bank sectors with a heavy policy burden and low efficiency of financial allocation weaken economic growth and worsen environmental degradation due to their inefficient allocation of loans in China. On the other hand, financial leverage that is measured by the ratio of share of credit to the private sector to the nominal GDP will increase with the slowing down of economic growth. Evidently, economic growth caused by financial leverage reacts on the financial leverage. Financial leverage will lead to economic and financial instability due to a vicious spiral of leverage and growth.

Second, economic growth impetuses for carbon emissions increase. This finding reveals that decades of economic growth in China was attained at the cost of natural resources and the environment. For instance, promoting heavy and polluting industries has contributed significantly to economic growth and carbon emissions.

Finally, financial leverage reacts positively and significantly to an innovation of carbon emissions. The bank sector has a strong credit preference toward heavy industries and bank credit is mainly utilized in carbon intensive sectors with state-owned enterprises benefitting from the policies enacted by the government in China. Over the years, this preferential treatment leads to credit precipitation in the inefficient state-owned sector, so as to increase financial leverage.

Several policy implications follow from the above analysis. It should be noted that China’s current financial system is bank-dominated, while by financial deleveraging, China’s financial system should be constantly enriched, especially giving financial support for efficient non-state-owned enterprises and environmentally-friendly projects. Meanwhile, excessive financial leverage has a restraining influence on economic growth, but economic growth can help lower the financial leverage ratio in China. Therefore, the government should be vigilant to financial deepening and development and tolerance of the financial leverage ratio rising appropriately to stimulate economic growth. Furthermore, our results cast a new light on the tight relationship among financial leverage, economic growth and environmental degradation in China, which suggest that China should deepen financial supply-side structure reform and improve the quality of financial resource use so as to help transform and upgrade real economy, which is conductive to developing a low-carbon economy. Therefore, we should pay more attention to the financial risk management and control of excessive or even higher financial leverage and establish a long-term mechanism for optimizing financial structure to perfect various financing channels and promote updating the industrial structure. Bank sectors should improve their ability to price risk and green credit supply while the government should promote green credit policies and clarify its own behavior boundaries to relieve the mismatch of financial resources.

## Figures and Tables

**Table 1:** Carbon emissions coefficients of various fossil fuels.

Fossil Fuel	Average Low Calorific Value (kJ/kg)	Carbon Content (kgC/GJ)	The Rate of Carbon Oxidation	Carbon Emissions Coefficients (tC/t)
Coal	20,908	25.8	0.913	0.4925
Coke	28,435	29.2	0.928	0.7705
Crude oil	41,816	20.0	0.979	0.8187
Fuel oil	41,816	21.2	0.985	0.8691
Gasoline	43,070	18.9	0.980	0.7977
Kerosene	43,070	19.5	0.986	0.8281
Diesel	42,652	21.1	0.985	0.8691
Natural gas	38,931(kJ/m^3^)	15.3	0.990	0.5896 (tC/m^3^)

**Table 2:** Variable description and data sources.

Variables	Description	Definition	Source
LEV	Financial leverage ratio	The ratio of private sector credit to nominal GDP (%)	China Statistic Yearbook
GDP	Economic growth	The GDP per capita (RMB) at constant 1997 price in log form	China Statistic Yearbook
$CO_{2}$	Environmental pollutions	The carbon dioxide per capita (mt) in log form	China Energy Statistical Yearbook, China Statistic Yearbook

**Table 3:** Description statistics.

Variables	Type	Mean	Std. dev.	Min	Max	Observations
LEV	Overall	1.09	0.33	0.53	2.27	N = 600
	Between		0.28	0.73	1.95	n = 30
	Within		0.19	0.45	2.02	T = 20
GDP	Overall	7.30	0.77	5.39	9.08	N = 600
	Between		0.49	6.34	8.45	n = 30
	Within		0.60	6.08	8.42	T = 20
$CO_{2}$	Overall	4.36	1.74	0.05	7.44	N = 600
	Between		1.25	1.74	6.44	n = 30
	Within		1.23	0.88	7.17	T = 20

Source: author’s own compilation.

**Table 4:** Results of panel unit root tests.

Variables	*LEV*	*GDP*	*CO*_2_	Δ*(LEV)*	Δ*(GDP)*	Δ*(CO_2_)*
IPS	6.785	0.0025	−5.6265 ***	−10.833 ***	−1.704 **	−8.747 ***
Fisher-ADF	35.648	58.395	112.496 ***	214.255 ***	79.533 **	181.186 ***
Fisher-PP	21.300	26.989	77.4799 *	318.171 ***	75.497 *	204.099 ***

Notes: ***, ** and * denote significance at the 1%, 5% and 10% level, respectively. Δ(⋅) indicates the first differences.

**Table 5:** Results of the PVAR model.

Response of	Response to					
	Δ*(LEV_t−1_)*	Δ*(LEV_t−2_)*	Δ*(GDP_t−1_)*	Δ*(GDP_t−2_)*	Δ*(CO_2t−1_)*	Δ*(CO_2t−2_)*
$\Delta (LEV_{t})$	0.127 ** (2.20)	−0.0.34 (−1.02)	−2.57 *** (−6.95)	0.018 (1.61)	0.035 *** (2.84)	0.007 (1.06)
$\Delta (GDP_{t})$	0.024 (1.56)	−0.011 ** (−2.10)	1.010 *** (12.26)	0.006 ** (2.18)	−0.001 (−0.29)	0.001 (0.73)
$\Delta (CO_{2t})$	−0.010 (-0.07)	−0.209 (−1.60)	2.509 ** (2.26)	0.001 (0.01)	0.108 * (1.82)	0.067 ** (2.22)

Notes: Reported numbers show the coefficients of regressing the row variables on lags of the column variables. z-statistics are in parentheses. ***, ** and * denote significance at 1%, 5% and 10%, respectively. Δ(⋅) indicates the first differences.

**Table 6:** Variance decomposition of the PVAR model (%).

Variables	Δ*(LEV_t_)*	Δ*(GDP_t_)*	Δ*(CO_2t_)*
$\Delta (LEV_{t})$	36.5	63.1	0.50
$\Delta (GDP_{t})$	6.90	93.1	0.00
$\Delta (CO_{2t})$	3.50	26.8	69.7

Notes: The results are based on the orthogonalized impulse-responses. Percent of variation in the row variable (10 periods ahead) is explained by the column variable. Δ(⋅) indicates the first differences.
